# Examination of factors causing postoperative pneumonia in elderly hip fracture patients: A narrative review

**DOI:** 10.1097/MD.0000000000041700

**Published:** 2025-02-28

**Authors:** Dongqi Ji, Huanhuan Li, Shasha Jin, Chunyan Tian, Liang Wu

**Affiliations:** a Beijing Xiaotangshan Hospital, Beijing, China; b Tianjin Key Laboratory of Exercise Physiology and Sports Medicine, Institute of Sport, Exercise & Health, Tianjin University of Sport, Tianjin, China.

**Keywords:** elderly hip fracture, independent factors, pathogenic bacteria, postoperative pneumonia, risk factors

## Abstract

With the increasing aging problem, the number of fractures in the elderly is also increasing, of which hip fractures are more common, known as “the last fracture of life.” Postoperative pneumonia (POP) is a common complication of hip fracture, which greatly increases the mortality of patients. It is particularly important to clarify the factors of perioperative pneumonia for the prevention and treatment process. In this paper, the factors causing POP mainly include demographic factors, pre-injury comorbidities, blood index parameters, major clinical interventions and related mechanisms were reviewed, and the risk degree of the factors causing postoperative pneumonia was mainly discussed, and they were divided into independent factors and risk factors. The objective is to make the most accurate POP prevention measures for hip fracture patients according to the classification of independent factors and risk factors, and reduce the incidence of postoperative pneumonia.

## 1. Introduction

The aging of society has been aggravated by advances in medical care and quality of life, and the decline in hormone levels with age has led to an increase in the number of osteoporosis in the elderly. The number of osteoporosis-induced fractures in the elderly has increased dramatically, with hip fractures being the most common, making them a common problem for the elderly worldwide.^[1,2]^ There are more than 10 million hip fracture patients worldwide each year, and it is expected to exceed 20 million by 2050.^[3,4]^ It is estimated that 20% to 60% of patients need help to meet daily life within 2 years after surgery, which seriously affects the quality of life of patients, and patients spend more than $40,000 in the first year after a hip fracture. The state needs to bear more than $17 billion in care costs every year, placing a great burden on families and society.^[3,5]^ The preferred treatment for patients with hip fracture is surgery, but postoperative complications are often the direct cause of death in hip fracture patients. Postoperative pneumonia (POP) is the most common.^[2]^ Epidemiological evidence shows that postoperative pneumonia significantly increases the 30-day mortality rate to 27% to 43%,^[6]^ so reducing the risk of POP and preventing and treating POP is the key to reducing the mortality rate of hip fracture patients. It has been reported that there are many factors affecting hip fracture POP, such as age, gender, respiratory diseases, operation time, etc. These factors are defined as related risk factors, and there is no study to classify the risk of these factors. This paper focuses on summarizing the factors affecting postoperative pneumonia in hip fracture patients and analyzing their severity, with the aim that different treatment protocols can be carried out in the clinic for independent factors and risk factors, and the importance of independent factors can be strengthened, so as to improve the quality of healthcare services and reduce the mortality rate of patients.

## 2. Method

This narrative review was conducted by collecting reviews of clinical trials, basic studies, and factors affecting postoperative pneumonia in patients with hip fracture. From the date of database establishment to March 2024, the following databases were searched in PubMed, Embase, Web of Science and Cochrane Library, including the following keywords: Factors of pneumonia after hip fracture, factors of pneumonia after intertrochanteric fracture of femur, factors of pneumonia after femoral neck fracture. The title and abstract of the study were independently checked by 2 authors (Dongqi Ji and Huanhuan Li.) and rigorously checked by 3 independent reviewers (Shasha Jin, Chunyan Tian, and Liang Wu). Conflicting views were discussed before a consensus was reached. We searched relevant articles by title and abstract and selected articles for further reading, and all included articles are discussed in detail in this review.

## 3. Types of postoperative pneumonia after hip fracture and distribution of pathogenic bacteria

At present, the most common types of pulmonary infection in clinical practice include acquired pneumonia, hypostatic pneumonia and aspiration pneumonia.^[7]^ Defining the risk factors of pneumonia in perioperative patients and investigating the distribution of causative bacteria are crucial for the prevention and treatment of POP, and the results of causative bacteria help to select appropriate antibiotic treatment. At present, some scholars have analyzed the pathogenic bacteria in hip fracture POP patients by clinical research, and pathogenic bacteria were detected from sputum analysis of patients with pulmonary infections in previous studies.^[8–11]^ Tang et al.^[8]^ analyzed the distribution of pathogenic bacteria in 32 hip fracture POP patients, and the results showed that gram-negative bacteria accounted for the largest proportion, and Klebsiella pneumoniae was the main infection bacteria, which was the most common infection bacteria in hip fracture POP patients. Next were gram-positive bacteria and fungi, mainly *Staphylococcus aureus* and *Candida albicans*. 43.8% of the patients were infected with 2 or more pathogens. Xiang et al^[11]^ isolated 153 different pathogenic strains from the sputum samples of patients diagnosed with POP after fracture surgery, among which 26 strains were multi-drug resistant. Currently, antibiotics are mainly used to treat pathogenic bacterial infections, but due to the increase of drug-resistant bacteria caused by the empirical use of antibiotics by healthcare workers in the clinic, the therapeutic efficacy of the treatment is significantly reduced.

## 4. Factors of postoperative pneumonia after hip fracture

There are many factors of pneumonia following hip fracture, including 4 main categories: demographic factors, pre-injury complications, blood indicators, and major clinical interventions.

### 4.1. Demographic factors

#### 4.1.1. Age

There is no conclusive evidence on the role of age as an independent risk factor for POP in postoperative hip fracture patients, and early literature attributed the risk of POP to the fact that as age increases, the elderly have more underlying diseases and because of hip surgery, their immunity is again lowered, thus increasing the likelihood of invasion of pathogens and the chances of POP, and not to a role of age per se.^[12]^ It has been shown that although the mortality rate is much higher in patients over 70 years of age than in patients under 70 years of age, the mortality rate is not differentially significant for patients in the same American Society of Anesthesiologists (ASA) classification group.^[13]^ A recent meta-analysis^[14]^ showed after multifactorial regression analyses that age was shown to be highly covariate or confounded with other risk factors, suggesting that increased comorbidities and other problems secondary to aging may be more responsible for POP. However, in recent years clinical studies have identified age as an independent risk factor for POP by most scholars after controlling and excluding some confounding factors, but there are different results in various studies on the inflection point of the age factor, Yu et al^[9]^ found a significant difference in age between the infected and uninfected groups by including 267 patients and the chances of developing the disease were significantly greater when the patient’s age was ≥70 years. The same results were obtained in another study^[15]^ and indicated that the incidence of POP decreased by 6.7% in patients over 70 years of age who underwent postoperative prophylaxis strategies; however, in 2 other studies^[16,17]^ it was indicated that the inflection point of this phenomenon was at the age of 80 and 90 years. Although it is still controversial whether the age factor can be used as an independent risk factor, it is certain that the probability of POP increases significantly with age, and advanced age should not be used as an absolute contraindication to surgery, and that patients with advanced age should be more concerned about their pulmonary status and be treated with appropriate targeted interventions in order to mitigate the risk of POP and reduce the mortality rate.

#### 4.1.2. Gender

Gender as an independent factor is still controversial; some studies^[14,16–18]^ have considered gender to be an independent risk factor for POP and have stated that males are the strongest independent risk factor. A cohort study by Jang et al^[18]^ showed that the prevalence of pneumonia in male patients was 16.39%, whereas the prevalence of pneumonia in female patients was only 9.29%; data showed^[18,19]^ that the incidence of postoperative pneumonia in men was 2.1 times higher than that in women, and its mortality rate was 1.74 times higher than that in women. Part of the reason may be related to the breathing pattern of men and women; women tend to breathe thoracically while men tend to breathe abdominally with less thoracic mobility. However it has been reported^[20]^ that the cause of the higher incidence of POP in men is that prior to surgery, men with hip fractures are already in poorer health and have a higher incidence of comorbidities than women, and are therefore at 2 times the risk of pneumonia, and patients in the POP group have been found to be more inclined to smoke.^[16]^ Male patients may have a more extensive history of smoking, which can alter the lungs by increasing the number of abnormal cilia cell biology and impair mucociliary clearance. When adjusted for potential comorbidities, there was no difference in the risk of dying from pneumonia between the 2 sexes.^[18]^ And Meyer et al^[19]^ elaborated that although the level of comorbidities and mortality was higher in male hip fracture patients than in females, the ASA score was equally predictive of complications in males and females. So the higher risk in male patients is not due to gender differences but due to potential comorbidities in male patients. Therefore in the case of hip patients, more attention should be paid to male history, daily habits, etc., and gender factors should be included in the consideration of risk factors for POP.

#### 4.1.3. American Society of Anesthesiologists

Grading: The American Society of Anaesthesiologists grading is a subjective assessment of a patient’s tolerance of surgery, which is divided into 6 grades according to the patient’s mobility and comorbidities, and can be used to assess the patient’s preoperative health status. Therefore, the difference in the incidence of POP in the clinical study due to age and gender may be due to the fact that the patients’ preoperative poor health status was greatly increased because of the comorbidities of the patients. Therefore, the difference in the incidence of POP due to age and gender in the clinical studies may be due to the fact that the patients’ preoperative health status was significantly higher due to poor health status due to comorbidities and other factors, which are not related to age or gender per se. A large number of studies have come to the consistent conclusion that the incidence of POP is significantly higher in postoperative hip fracture patients with ASA ≥ grade 3.^[21–24]^ Meyer et al^[21]^ reported that ASA scores can be used to predict various complications in postoperative hip fracture patients. Xiang et al^[22]^ statistically analyzed 166 patients with POP, of which 82.6% had pneumonia. 82.6% of these patients with pneumonia had ASA ≥ grade 3 and only 17.4% had ASA < grade 3. The study showed a progressive increase in the risk of postoperative mortality with increasing grade, with individuals with an ASA score of 3 being almost 4 times more likely to develop pneumonia than the reference group.^[21]^ Although the ASA is easier to perform as a routine assessment, its limitations still need to be considered, namely the high subjectivity in assigning scores and the presence of low to moderate inter-rater reliability, and therefore other factors still need to be considered in the clinical setting in conjunction with the ASA thereby preventing the occurrence of POP. Further studies on ASA are needed in the future with the aim of improving its value as a prognostic indicator for postoperative complications.

#### 4.1.4. Body mass index

One study^[25]^ found a negative correlation between body mass index and POP, where patients in the aspiration pneumonia group had a mean index of 19.6 kg/m^2^ compared to 22.2 kg/m^2^ in the control group, and the same result was statistically found in another study, where patients in the nonpneumonia group had a mean increase in body mass index (BMI) of 3.5 when compared to the pneumonia group. Part of the reason may be that elderly people have swallowing dysfunction, which leads to low BMI and malnutrition, increasing the risk of inflammation. According to Bohl et al^[17]^ the risk of POP was greatest at BMI < 18.5 kg/m^2^ and least at BMI > 30 kg/m^2^. However, some scholars have excluded BMI as an independent risk factor, and the reason for the difference in results may be due to the difference in BMI, as people with low BMI are more prone to fracture, which leads to a high proportion of low BMI in the population with fracture, resulting in a reduced sample size of high BMI, which affects the statistical results. And there are fewer studies on the correlation between BMI and POP, and the mechanism is still unclear, so further studies are still needed to verify the correlation.

#### 4.1.5. Lifestyle habits

Patients with poor lifestyle habits such as smoking, alcohol abuse and physical inactivity should also be considered as risk factors.^[11,14]^ There are fewer studies on the correlation between unhealthy lifestyle and POP, but it has been reported that a history of smoking can be an important factor in POP.^[14]^ Harmful substances such as carbon monoxide and nicotine in patients with a history of smoking can reduce the clearance capacity of the cilia, leading to an increase in secretions in the lungs, resulting in pulmonary fibrosis or even cancer, and increasing the likelihood of postoperative pneumonitis.^[26,27]^ Long-term alcohol consumption will aggravate the detoxification burden of the liver, reduce the body’s detoxification ability, and also cause corresponding damage to the cardiovascular system, lowering the immune system.^[28,29]^ Elderly patients who lack exercise have reduced motor function and decreased immune function, exacerbating their own frailty, and studies have shown that the risk of POP increases 3.32 times in patients with mild frailty and 5.36 times in patients with severe frailty.^[30]^ Therefore, patients should be questioned about their bad living habits at the time of admission, and such patients should be strengthened with supervision and education to persuade them to quit smoking and drinking to reduce the occurrence of POP.

### 4.2. Pre-injury comorbidities

The type and number of pre-injury comorbidities also tends to have a direct impact on the incidence of POP in patients, which is largely attributed to poorer pre-injury status, which tends to exacerbate comorbidities after surgery is performed. The types of comorbidities reported include respiratory disease, diabetes mellitus, cerebrovascular disease, dementia, coronary artery disease, chronic renal failure, cardiac arrhythmia, and heart failure, etc. Zhang et al^[24]^ concluded from a multifactorial analysis that individuals with at least 2 comorbidities accounted for 72.9% of patients with POP, and LV^[12]^ conducted multivariate logistic regression analysis on 1429 patients who underwent hip surgery and showed that the number of comorbidities ≥ 3 was positively correlated with the incidence of POP.

#### 4.2.1. Respiratory diseases

Numerous studies have shown^[24,31]^ that respiratory diseases can be an independent risk factor for POP. According to a retrospective cohort study,^[32]^ respiratory diseases were significantly correlated with the incidence of POP (*P* < .01), and the incidence of POP in patients with respiratory problems was 5 times higher than that in the general population.^[33]^ The body of chronic obstructive pulmonary disease patients is in a state of chronic inflammation and immune system disorders, accompanied by up-regulation of C-reactive protein and increase in inflammatory cytokines and tissue factors.^[34]^ Moreover, hip fracture patients suffer from weaker gas exchange due to reduced immunity postoperatively and lower cilia clearance, thus reduced lung function, leading to POP. Dyspnoea on exertion and asthma have also been cited in the literature as risk factors for POP in hip fracture patients.^[17,35]^ Targeted interventions for patients with respiratory disease are therefore essential in the prevention and treatment of POP and can be optimized through programmes such as elevation of the head of the bed, early mobility and pain control.

#### 4.2.2. Diabetes mellitus

Diabetes mellitus can also be an independent factor for POP in hip fracture patients. A study^[36]^ compared patients with diabetes mellitus by comorbidities with patients without diabetes mellitus and found that patients with comorbidities had statistically significantly higher probability of POP. However, some studies^[32]^ showed no significant difference between diabetic and non-diabetic patients and was not a risk factor for POP. Tang et al^[37]^ determined that the incidence of POP was significantly higher in patients with hyperglycemia than in patients with normal blood glucose levels by using multifactorial logistic regression and propensity score matching analyses in 600 patients and found that compared to diabetic patients, nondiabetic patients were found to be at greater risk of POP when their blood glucose was elevated. Therefore, perhaps elevated blood glucose is the most important factor of POP and is more severe in the postoperative period. Related studies have shown that there are several mechanisms by which elevated blood glucose increases the risk of POP. (i) It may be through stress-induced hyperglycemia leading to enhanced oxidative stress, resulting in the production of harmful molecules such as oxygen and nitrogen free radicals in lung tissue, which can damage the structure and function of lung tissue.^[37]^ (ii) Elevated blood glucose levels can bind to complement factors and glycosylated immunoglobulins and complement, decreasing the body’s immune competence, thus the body’s ability to clear bacteria is weakened and the risk of POP is increased.^[31,38]^ (iii) Stress-induced hyperglycemia also triggers an inflammatory response, inducing the production of inflammatory cytokines, which further exacerbates lung inflammation.^[39]^ Although there is no consensus on the relationship between diabetes mellitus and POP, its ability to act as an immune-mediated chronic inflammatory disease remains a significant risk. Therefore, perioperative glycemic control is essential for patients with a history of diabetes; for patients with normal blood glucose, close attention should be paid to changes in blood glucose to prevent sudden rises in blood glucose, thereby reducing the incidence of POP.

#### 4.2.3. Preoperative dependency status

Whether or not a hip fracture patient’s pre-injury functional status was independent has also been suggested to be an important influencing factor for POP. Zhang et al by including 1409 patients in their analysis showed that the incidence of POP in patients with preoperative dependency was 2.21 times higher than that of patients with preoperative independent status; the proportion of patients with functional status dependency in the pneumonia group was reported to be significantly higher than that of the non-pneumonia group (53.6%:12.6%).^[22]^ Preoperative dependent status is often due to cognitive dysfunction or motor dysfunction caused by complications in the patient, such as dementia or stroke. Some studies^[25,33]^ have included dementia and stroke among the independent factors of POP in hip fracture patients, and a more reasonable explanation is that patients with dementia and stroke are in a long-term dependent state due to dysfunction, and that improper feeding by the patient’s family members and the patient’s own bed-ridden factors may increase the risk of aspiration, and that the respiratory muscles’ strength is also weakened, and coughing and expectoration are reduced, thus Pneumonia is triggered, coupled with the patient’s own decreased immunity, the sensitivity of POP is again increased. However, the relationship between patients’ dependence status and POP is less well studied and the mechanism is not clear, so it can only be used as a reference for POP susceptibility factors in clinical practice.

#### 4.2.4. Other comorbidities

Most scholars can basically agree on respiratory diseases and diabetes mellitus as independent factors of POP in hip fracture patients. However, the relevance of comorbidities such as cardiovascular disease, chronic renal failure, hypertension, and cancer to POP has been described in some recent literature.^[24,31,32,40]^ Cardiovascular diseases^[32,41]^ may cause hemodynamic disturbances, resulting in pulmonary congestion and edema, or decreased function of the lung organs due to reduced cardiac function, with a significant decrease in lung capacity and ventilation, increasing the risk of pneumonia. Patients with renal insufficiency are prone to hypoproteinaemia and malnutrition, as well as accumulation of metabolites, such as hydrogen ions, histamine and serotonin, leading to acidosis and other disturbances of the internal environment, resulting in abnormalities in humoral cellular immunity.^[42]^ Most studies consider these comorbidities as risk factors for POP, and a recent meta-analysis^[34]^ summarized the results to the same conclusion; however, due to the small number of studies or sample sizes of these factors, and the fact that the mechanisms influencing the occurrence of POP have rarely been explored, studies are still needed to clarify the independence of the various comorbidities as well as the mechanisms. However, it is certain that the greater the number of comorbidities, the greater the incidence of POP, and in general, coexisting somatic diseases are immutable. Therefore, clinicians should have detailed information about coexisting conditions in order to assess the risk of POP and to identify high-risk patients for preventive strategies.

### 4.3. Blood indicators

#### 4.3.1. ALB

Serum albumin (ALB) is the most commonly used serum marker in the clinic for assessing protein-energy malnutrition in hip fracture patients. A preoperative albumin level below 35 g/L is considered malnutrition and below 30 g/L is identified as hypoalbuminaemia. It was found^[43]^ that the reason for preoperative hypoalbuminaemia to be an independent factor leading to POP may be that hip fractures and muscle weakness require a large amount of protein for recovery, therefore protein deficiency leads to slower muscle recovery and prolonged fracture healing time, increasing the patient’s time in bed, thus raising the risk of POP in patients. In addition, low ALB levels lead to decreased plasma colloid osmolality and increased interstitial fluid volume, which may further develop into pleural and pulmonary infections. Wang et al^[31]^ found that the risk of POP in patients with preoperative hypoalbuminaemia was 5.187 times higher than that of patients with normal preoperative albumin levels by including 720 patients in their study. Most of the scholars have studied preoperative hypoalbuminaemia more and have identified it as an independent factor. In recent years, a study^[40]^ analyzed the prevalence of postoperative pneumonia by using inverse treatment probability weighted and propensity score matched analyses in 1155 patients and found that postoperative hypoalbuminemia was also an independent factor for POP in hip fracture patients. Hypoalbuminaemia may suppress the innate immune response by promoting granuloma formation and reducing collagen synthesis, thereby predisposing patients to infections and other postoperative complications. These highlight postoperative immune dysfunction, manifested by early postoperative hypoalbuminaemia, which may lead to the development of postoperative pneumonia. Therefore, patients undergoing femoral neck fracture surgery should have their ALB levels routinely measured on admission and after surgery, and patients with preoperative and postoperative hypoalbuminaemia should receive intensive monitoring and intensive perioperative care, with nutritional status corrected by the administration of high-protein supplements before proceeding to surgery.

#### 4.3.2. Hemoglobin

The main function of hemoglobin (Hb) is to transport oxygen, so Hb is closely related to respiration. The World Health Organization defines anemia as <130 g/L in men and 120 g/L in women, anemia reduces the body’s immunity and exacerbates the possibility of inflammation.^[44]^ A large number of studies^[12,17,34]^ have pointed out that low hemoglobin is can be an independent risk factor for POP in hip fracture patients, and it has been reported^[45]^ that patients with combined anemia have a 2.84-fold increase in the incidence of POP, however, the latest studies have pointed out that the relationship between hemoglobin level and POP is nonlinear, and that when the level of Hb is lower than 83.5 g/L, for every 1 g/L increase in Hb, the POP was associated with a 9% reduction in the risk of POP, however, on the right side of the inflection point, the incidence of POP did not change significantly with increasing Hb levels.^[46]^ Therefore, optimizing the perioperative treatment and care of anemic patients is essential to reduce the incidence of POP after hip fracture surgery, with the preoperative period being the best time to improve anemia.^[44,47]^ The decision to administer a blood transfusion should be made on an individual basis and the potential risks and benefits of such an intervention should be considered.

#### 4.3.3. Red cell distribution width

Red cell distribution width (RDW) is a measure of changes in red blood cell size and in recent years has been found to be an independent predictor of infectious and inflammatory conditions, studies have shown a correlation between RDW and POP, Lv et al^[12]^ concluded from a prospective study that for every 1/4 increase in RDW, the risk of POP would increase by 1.459 times. Risk will increase by 1.459 times. However, the 2 were not positively correlated, and an inflection point in the correlation between the 2 was reported, with a positive correlation when RDW < 14.3%, a 61% increase in incidence for every 1% increase in RDW, and saturation when RDW > 14.3%.^[48]^ The same conclusion was obtained in another study and this inflection point was considered to be 14.5%. The possible reason for the correlation between preoperative RDW and the incidence of POP is that increased production of pro-inflammatory cytokines may be responsible for the inflammatory response, leading to an increase in peripheral blood erythrocyte malmaturation and naïve erythrocytes, which in turn leads to an increase in RDW.^[49]^ Therefore RDW needs to be considered as an independent risk factor in clinical practice.

#### 4.3.4. Other blood indicators

Other positive correlations have been reported in the literature, such as C-reactive protein, platelets, leukocytes, creatinine, arterial blood partial pressure of oxygen (PaO2), D-dimer, creatine kinase, D-dimer, creatine kinase, b-type natriuretic peptide, and calcitoninogen.^[12,22,50–52]^ The results showed that thrombocytopenia on admission, PaO2 < 72.5 mm Hg, b-type natriuretic peptide threshold > 75 ng/L, D-dimer > 2.26 mg/L can be considered as independent risk factors for POP, and high serum creatinine has been reported to increase the risk of POP by 3.289 times. A plausible explanation is that all these indicators are related to the patient’s immune system, and a reduced immunocompetence is associated with a significantly higher risk of POP. In recent years, the detection of inflammatory/immune markers has been applied in the clinic after fracture surgery to respond to the inflammatory state of the body and cell-mediated immunity, which is used to predict the likelihood of POP, and its sensitivity to POP is higher than that of traditional blood markers, such as neutrophils/lymphocytes (NLR) and platelets/lymphocytes (PLR), which was found to be associated with NLR > 5.84.^[53]^ POP had a significant correlation and could be considered as independent factors. However, these indexes are not well documented due to fewer studies, unclear mechanisms, and insufficient evidence, and continued studies are needed to explore the correlation between the indexes and POP.

### 4.4. Clinical intervention factors

The main clinical interventions in terms of factors include delay in surgery, duration of surgery, mode of anesthesia and mode of surgery are all risk factors for POP in postoperative hip fracture patients.

#### 4.4.1. Delay in surgery

Delay in surgery is the prolongation of time between injury and surgery, most scholars now believe that surgery before it is too late is the least risky and greatly reduces mortality, the threshold time for delaying surgery is usually defined as 6 to 72 hours, with 48 hours being the most common threshold time. Xiang et al^[22]^ counted 1113 patients and compared to the pneumonia group, the proportion of patients in the non-pneumonia group with injury to surgery ≥ 48 hours was significantly lower (84.2% versus 46.2%); Tian et al^[32]^ found an 8% increase in the risk of postoperative pneumonia for every day surgery was delayed. The reason for the increased risk of POP due to delayed surgery may be because delayed surgery leads to persistent pain and thus braking, increased time in bed, which weakens the ability to cough and expectorate, and an increased risk of fallout pneumonia. Therefore, surgery should be scheduled as soon as possible to reduce the occurrence of POP. Domestic experts in the diagnosis and treatment of geriatric hip fractures have reached a consensus on the optimal time for surgery, stating that surgery should be performed within 24 h to 48 h of the fracture.^[54]^ Factors leading to surgery may include the patient’s own poor condition or the limitation of surgical conditions in healthcare institutions, so healthcare institutions should optimize the emergency measures to achieve patients to be operated within 48 h or even 24 h to reduce the occurrence of bed-dependent complications.

#### 4.4.2. Duration of surgery

In recent years it has been suggested that the duration of surgery also affects the incidence of POP. Previous studies have shown a positive correlation between the duration of surgery for hip fracture patients and complications and mortality rates.^[55]^ Byun et al^[25]^ who analyzed the duration of surgery for patients with pneumonia and non-pneumonia found that the duration of surgery for the patients in the pneumonia group was 92 min, whereas it was only 71.4 min for the non-pneumonia group, which was a significant difference between the 2 groups (*P* < .001). Prolonged surgical time often causes is excessive bleeding, patients’ body temperature to drop, which in turn causes the patient’s immunity to decline, increasing the risk of lung infection. Studies have shown that the time for surgery to be performed > 120 min is considered an independent risk factor for POP in hip fracture patients.^[9]^ Factors contributing to prolonged surgery may come from the patients themselves, such as severe osteoporosis or complex fracture patterns, or perhaps from inadequate surgeons and medical equipment. However, a recent meta-analysis^[14]^showed that the difference between the duration of surgery on POP was not significant. The relationship and mechanisms between duration of surgery and POP have been less studied in clinical practice, although minimizing the duration of surgery and establishing perioperative management strategies (e.g. blood transfusion) are still important to prevent the development of aspiration pneumonia.

#### 4.4.3. Mode of anesthesia

Some scholars^[9,32]^ believe that the choice of anesthesia mode during surgery for patients with hip fracture also affects the occurrence of POP. Anaesthesia methods in clinical practice are mainly general anesthesia and local anesthesia. Tian et al^[32]^ showed that the risk of POP in patients undergoing general anesthesia was 1.61 times higher than that in patients undergoing local anesthesia. This may be due to the fact that general anesthesia, as an invasive procedure, indirectly affects the function of respiratory muscles, and the need for tracheal intubation in such patients, which prevents patients from breathing on their own and stimulates the production of respiratory secretions, thus increasing the likelihood of pneumonia. However, in a previous study^[56]^ it was shown that although general anesthesia causes an increased risk of in-hospital mortality and readmission in patients, there was no significant correlation with postoperative pneumonia. There are fewer studies on the choice of anesthesia modality affecting POP and the evidence for the effect of anesthesia modality on pneumonia is inconclusive; anesthesia modality can only be considered as a factor. The choice of anesthesia can only be considered as a factor, as the patient’s physical condition, the family’s choice, and the physician’s preference can all lead to changes in anesthesia, and therefore the choice of anesthesia needs to be communicated to multiple parties before it can be determined.

#### 4.4.4. Other

The primary treatment for patients with hip fractures is currently surgery, and studies have shown^[53]^ that the surgical procedure also affects the development of POP, with patients undergoing intramedullary nail fixation having the highest risk of pneumonia, and patients with intertrochanteric femoral fractures being at higher risk than patients with neck of femur and proximal femur fractures. Mitochondrial DNA has been reported to be an activator of inflammation and the innate immune system, and intramedullary nailing surgery accelerates the release of mitochondrial DNA, exacerbating the systemic inflammatory response, as well as lung injury in elderly hip fracture patients.^[57]^ However, there are also different conclusions about the high mortality rate of patients undergoing total hip arthroplasty.^[58]^ Mechanical ventilation for more than 24 hours and postoperative bed rest for more than 3 days have also been suggested as independent factors for POP. However, since there are relatively few studies in this area and the choice of surgical procedure is based on the degree of injury, type of injury, etc, comprehensive consideration should be conducted to study the association between the 2.

## 5. Conclusion

Due to the accelerating aging process, the proportion of elderly people is increasing, making healthcare for the elderly a priority for society. For older adults with hip fractures, prevention and treatment of POP and reduction of disability and mortality are key for healthcare providers. Therefore, in the face of perioperative hip fracture patients, it is important to take proactive medical treatment to deal with the occurrence of POP both preoperatively and postoperatively, and to explore the factors that lead to the development of pneumonia in patients, so as to personalize the prevention of POP. This paper mainly summarized independent factors and risk factors affecting postoperative pneumonia in patients with hip fracture, and pointed out the correlation, mechanism and unclear points of influencing factors (see Table 1). At present, there is no literature to fully indicate the influencing factors and related mechanisms of postoperative pneumonia in patients with hip fracture. Independent factors included ASA grade, respiratory disease, number of comorbidities, preoperative dependence status, preoperative ALB, hemoglobin, RDW and surgical delay. Independent factors were strongly correlated with the risk of POP, and special prevention programs should be carried out in clinical practice to reduce mortality. The risk factors mainly include age, BMI, unhealthy living habits, diabetes, postoperative serum protein, operation time and anesthesia method, etc, More attention should be paid to the risk factors. However, the mechanisms of some independent factors are still unclear, and the correlation between some risk factors and postoperative pneumonia cannot be determined. Further clinical research is still needed to determine the correlation between various factors and POP and the mechanism of influence, and continue to explore new treatment options on the basis of the current commonly used therapeutic measures (e.g., medication, respiratory training, etc).

**Table 1:** Factors influencing postoperative pneumonia in elderly patients with hip fractures.

Categorization	Specific factors	Degree of danger	Relevance	Ambiguity
Demographic factors	Age	Risk factor	The older you are, the higher the risk	Associated with comorbidities Age inflection point not yet determined
	Genders	Risk factor	Higher for males than females	Associated with comorbidities, smoking history
	ASA classification	Independent factor	ASA ≥ grade 3, significantly higher risk	none
	BMI	Risk factor	The lower the BMI, the higher the risk	Less well researched and the mechanisms involved are not clear
	habits	Risk factor	Unhealthy lifestyle habits increase the risk of pneumonia	Less well researched and the mechanisms involved are not clear
Pre-injury comorbidities	Respiratory diseases	Independent factor	COPD is the most typical	Other types of respiratory diseases are less well studied
	Number of comorbidities	Independent factor	Number of comorbidities ≥ 3, significantly higher risk	none
	Diabetes	Risk factor	uncertain	May be due to elevated blood sugar, not diabetes
	Preoperative dependency status	Independent factor	The higher the dependency, the greater the risk	Mechanisms are not yet clear
	Other comorbidities	Risk factor	e.g. cardiovascular disease, hypertension, chronic renal failure, etc. have a correlation with	Less well researched and the mechanisms involved are not clear
Blood indicators	Preoperative serum albumin	Independent factor	Malnutrition or low preoperative serum albumin, elevated risk	none
	Postoperative serum albumin	Risk factor	Low postoperative serum albumin, elevated risk	Fewer studies, insufficient evidence
	Hemoglobin	Independent factor	Low hemoglobin levels, increased risk	Whether the correlation is linear and whether there is an inflection point
	RDW	Independent factor	Elevated RDW, elevated risk, but not linearly so	Inflection point not yet clear, fewer studies
	Other blood indicators	Risk factor	Such as CRP, PaO2, BNP, D-dimer, etc. possess correlations	Less well researched and the mechanisms involved are not clear
Clinical Intervention Factors	Delay in surgery	Independent factor	The longer the delay, the greater the risk	none
	Surgical time	Risk factor	The longer it takes, the higher the risk	Less well researched and the mechanisms involved are not clear
	Anesthesia	Risk factor	Higher risk of general anesthesia	Fewer studies, insufficient evidence
	Other than	Risk factor	Associated with surgical approach, duration of mechanical ventilation	Less research

ALB = serum albumin, ASA = American Society of Anesthesiologists, BMI = body mass index, BNP = b-type natriuretic peptide, COPD = chronic obstructive pulmonary disease, CRP = C-reactive protein, Hb = hemoglobin, PaO2 = arterial blood partial pressure of oxygen, RDW = red cell distribution width.

## Author contributions

**Conceptualization:** Dongqi Ji, Liang Wu.

**Supervision:** Dongqi Ji, Huanhuan Li, Liang Wu.

**Visualization:** Dongqi Ji, Shasha Jin, Chunyan Tian.

**Writing – original draft:** Dongqi Ji, Huanhuan Li, Shasha Jin, Liang Wu.

**Writing – review & editing:** Dongqi Ji, Huanhuan Li, Shasha Jin, Chunyan Tian, Liang Wu.
